# Novel approach to synthesize NiCo_2_S_4_ composite for high-performance supercapacitor application with different molar ratio of Ni and Co

**DOI:** 10.1038/s41598-019-50165-5

**Published:** 2019-09-23

**Authors:** S. K. Shinde, Sivalingam Ramesh, C. Bathula, G. S. Ghodake, D.-Y. Kim, A. D. Jagadale, A. A. Kadam, D. P. Waghmode, T. V. M. Sreekanth, Heung Soo Kim, P. C. Nagajyothi, H. M. Yadav

**Affiliations:** 1 Department of Biological and Environmental Science, Dongguk University-Ilsan, Biomedical Campus, Goyang-si, Gyeonggi-do 10326 South Korea; 2 Department of Mechanical, Robotics and Energy Engineering, Dongguk University, Seoul, 04620 South Korea; 3 Division of Electronics and Electrical Engineering, Dongguk University, Seoul, 04620 South Korea; 4 Center for Energy Storage and Conversion, School of Electrical and Electronics Engineering, SASTRA Deemed University, Thanjavur, 613401 Tamilnadu India; 5 Research Institute of Biotechnology and Medical Converged Science, Dongguk University, Biomedi Campus, Ilsandong-gu, Goyang-si, Gyeonggi-do 10326 South Korea; 6 Department of Chemistry, Sadguru Gadage Maharaj College, Karad, 415124 India; 7 College of Mechanical Engineering, Yeungnam University, Gyeongsan, 48135 South Korea; 8 Department of Energy and Materials Engineering, Dongguk University, Seoul, 04620 South Korea

**Keywords:** Supercapacitors, Electrocatalysis

## Abstract

Here, we developed a new approach to synthesize NiCo_2_S_4_ thin films for supercapacitor application using the successive ionic layer adsorption and reaction (SILAR) method on Ni mesh with different molar ratios of Ni and Co precursors. The five different NiCo_2_S_4_ electrodes affect the electrochemical performance of the supercapacitor. The NiCo_2_S_4_ thin films demonstrate superior supercapacitance performance with a significantly higher specific capacitance of 1427 F g^−1^ at a scan rate of 20 mV s^−1^. These results indicate that ternary NiCo_2_S_4_ thin films are more effective electrodes compared to binary metal oxides and metal sulfides.

## Introduction

The development of sustainable electrochemical energy conversion methods and storage has sparked the interest of researchers aiming to produce devices that offer high power output, a long lifetime, and a short charging time to meet the increasing demand for power in daily life^1,2^. Supercapacitors have emerged as a promising energy storage device in this respect, with outstanding properties that include a high power density, a long cycle life, short response, rapid charging times, moderate energy density, modest maintenance requirements, and safe operation^2–5^. However, existing supercapacitor electrodes are mainly composed of activated carbon, binders, and conductivity enhancers, thus it is difficult to develop simple, lightweight supercapacitors. In general, supercapacitor performance depends mainly on the properties of the materials and synthesis methods used. Besides, carbon-based materials such as activated carbon, carbon nanotubes, and graphene exhibit low capacitance due to their surface dominant electrochemical double-layer storage mechanism^2^.

In recent years, significant research progress has been made on improving supercapacitor performance via the fabrication of nickel-cobalt-sulfide nanostructured electrode materials due to their higher electronic conductivity, strong redox reactions, high theoretical capacity, high cycling stability, variable oxidation states, environmental benign nature, easy and low preparation cost^6^. Nanomaterial composed of ternary metal sulfides with various structural morphologies have been applied as high performance pseudo supercapacitor electrodes, such as nanosheets arrays^2^, nanotubes^7^, nanorods^8^, urchins^9^, nanosheets^10^, hallow spheres^11,12^, nano-buds^13^, and flowers^14^. Ternary sulfides such as nickel cobalt sulfides have unique physical, chemical, and electrochemical properties, such as high specific capacitance, good electrochemical stability, and higher electronic and electrical conductivity compared to their oxide counterparts and binary sulfides^15–17^. In addition, the combination of Co and Ni in bimetallic sulfides leads to a higher redox potential and enhanced electrochemical energy storage performance compared with monometallic sulfides^9,18^.

Recently, Wei *et al*.^11^ synthesized hierarchically porous NiCo_2_S_4_ core-shell hollow spheres using the self-template method, which represents a one-pot solvothermal approach to the synthesis of hierarchical Ni-Co solid sphere precursors, followed by conversion to hierarchically porous NiCo_2_S_4_ core-shell hollow spheres via sulfidation treatment. Their core-shell hollow spheres depicted a specific capacitance of 1870.2 F g^−1^ at 2.0 A g^−1^ and excellent long-duration cycling. Shahrokhian *et al*.^17^ also reported a simple and efficient method for fabricating ternary metal sulfide electrodes based on the electrodeposition of nickel cobalt iron sulfide (Ni-Co-Fe-S) ultrathin nanosheets on the surface of 3D nickel nanocone arrays. The ternary metal sulfide electrode exhibited a high specific capacitance of 2159.7 F g^−1^ at 7 A g^−1^ with excellent rate capabilities. Lei *et al*.^19^ prepared NiCo_2_S_4_ nanosheets on carbon sponge using a hydrothermal method, leading to enhanced conductivity and ideal structural integrity. Their composite electrode delivered a specific capacitance of 1093 F g^−1^ at 0.5 A g^−1^ in a three-electrode system. Tao *et al*.^20^ developed a hierarchical Ni-Co-S nanosheets array based on a metal–organic framework on an Ni-foam electrode for supercapacitor applications. This nanosheets array system delivered rapid electron transportation, a short ion diffusion path, abundant active sites, and rich redox reactions. The electrode exhibited an electrochemical capacitance of 1406.9 F g^−1^ at 0.5 A g^−1^. Sun *et al*.^21^ reported the hydrothermal synthesis of hierarchical Ni-Co-S@Ni-W-O core–shell nanosheets arrays on nickel foam, producing a high specific capacitance of 1988 F g^−1^ at 2 A g^−1^.

The electrochemical performance of Ni-Co-S is influenced by several factors. For example, Jiang *et al*.^22^ synthesized porous NixCo_3−x_S_4_ (x = 0, 1, 1.5, 2, and 3) nanoparticles with various compositions of Ni–Co–S. The Ni_1.5_Co_1.5_S_4_ sample produced the highest specific capacitance (1093 F g^−1^ at 1 A g^−1^) in a three-electrode system. In contrast, Gao *et al*.^23^ reported the preparation of Ni_3−x_Co_x_S_4_ (x = 1.5, 2, 2.25, and 2.5) nanotube arrays on carbon cloth with different Co/Ni molar ratios. Ni_0.75_Co_2.25_S_4_ demonstrated a capacitance of 1856 F g^−1^ at 1 A g^−1^. Similarly, Chen *et al*.^24^ reported the fabrication of sea urchin-like Ni–Co sulfides with different ratios of Ni/Co, in which Ni_0.25_Co_0.75_S delivered the highest specific capacitance (676 C g^−1^ at 1 A g^−1^) in a three-electrode system.

Though the fabrication of NiCo_2_S_4_ nanostructures has been well researched to date, the synthesis of NiCo_2_S_4_ with specific hierarchical structures requires further investigation. In this sense, the development of Ni-Co-S electrode materials with varying compositions of Ni and Co is crucial to achieve optimal supercapacitor properties, such as high electrical conductivity, a porous structure, large capacitance, and excellent electrochemical stability. In the present study, we report the facile synthesis of Ni-Co-S (NCS) flake-like nanostructures on Ni mesh for supercapacitor applications. The effect of varying the composition of Ni and Co in the NCS nanostructures was also studied because the electrochemical performance of NCS electrodes can be enhanced by altering the molar ratio of Ni to Co precursors.

## Experimental Details

### Materials

Nickel nitrate hexahydrate (Ni(NO_3_)_2_.6H_2_O), cobalt nitrate hexahydrate (Co(NO_3_)_2_.6H_2_O), sodium sulfide nonahydrate (Na_2_S.9H_2_O), pottasium hydroxide (KOH) and ammonium hydroxide (NH_4_OH) were procured from Sigma Aldrich and used without futher purification.

### Synthesis of NiCo_2_S_4_ thin films

The fabrication process for nanoflakes-like NiCo_2_S_4_ thin films using the successive ionic layer adsorption and reaction (SILAR) method on Ni mesh is represented in Fig. S1a. SILAR is simplistic, economically feasible and most useful method in order to growth of material directly on the conducting and non-conducting thin films. Furthermore, SILAR method holds a potential to improve different surface morphologies by monitoring simple preparative parameters. To fabricate the basic form of this NCS nanostructure with a Ni to Co molar ratios of 40:10 (NCS:40), 0.05 M Ni(NO_3_)_2_.6H_2_O, 0.1 M Co(NO_3_)_2_.6H_2_O and 0.1 M Na_2_S.9H_2_O were each dissolved in 100 mL of double-distilled water (DDW) and pH was adjusted to 12 by addition ammonia solution. First, the flexible Ni mesh was immersed in a Ni(NO_3_)_2_ bath for 20 s to allow the Ni^2+^ ions to be adsorbed on the surface of the mesh. The Ni mesh was then cleaned in DDW for 5 s to remove any loosely bound Ni^2+^ ions. Following this, the Ni^2+^ adsorbed Ni mesh was placed in a Co(NO_3_)_2_.6H_2_O bath for 20 s to allow the adsorption of Co^2+^ ions onto the surface. The mesh was then washed with DDW to remove loosely bound Co^2+^ ions. In the final step, the Ni^2+^/Co^2+^ deposited flexible Ni mesh was immersed in the Na_2_S precursor solution where S^2−^ ions from the solution reacts with Ni^2+^ and Co^2+^ ions to form mixed metal sulfide film. Furthermore, it is rinsed in the DDW for 5 s to remove loosely bound S^2−^ ions. This process was repeated for 6 SILAR cycles to achieve appropriate film thickness^25,26^. In this work, different molar ratios between Ni to Co precursors have been considered (40:10, 30:20, 25:25, 20:30, and 10:40, referred to as NCS:40, NCS:30, NCS:25, NCS:20, and NCS:10, respectively).

### Characterization techniques

X-ray diffraction analysis of the prepared thin films was performed using a Rigaku Ultima III diffractometer operated at 40 kV and 40 mA with Cu K_α_ radiation (1.54 A°) as a source, with a scanning range of 2θ over 20–80°. Step-scan mode was applied with a step width of 0.02°, a sampling time of 1 s, and a measurement temperature of 25 °C. The chemical states of the elements present in the thin films were investigated by X-ray photoelectron spectroscopy (XPS; ULVAC-PHI Quantera SXM). The morphology of the samples was investigated via field emission scanning electron microscopy (FE-SEM) using a JEOL JSM-7100. The nanostructures of the prepared samples were visualized by high-resolution transmission electron microscopy (TEM; JEOL, Model JEM-2100).

### Electrode preparation and electrochemical measurements

Electrochemical performance was evaluated using a Versa Stat 3 (Princeton Applied Research) workstation by measuring cyclic voltammetry (CV), galvanostatic charge/discharge, and electrochemical impedance. A reference electrode probe was connected to an Ag/AgCl electrode and a counter-electrode probe was connected to thin platinum foil. A working electrode probe attached to the NCS/Ni mesh electrode and immersed in a 5 M KOH electrolyte solution. A cyclic potential sweep was applied with initial and final voltages of −0.4 and 0.6 V, respectively. Electrochemical impedance measurements were taken between 1 Hz and 100 kHz with an AC amplitude of 10 mV and a bias potential of 0.4 V.

## Results and Discussion

### Formation of NiCo_2_S_4_ thin films

NiCo_2_S_4_ thin films were synthesized by dipping a substrate into aqueous solutions of Ni(NO_3_)_2_.6H_2_O, Co(NO_3_)_2_.6H_2_O, and Na_2_S.9H_2_O separately. SILAR process is mainly based on ion by ion deposition, which exhibits the deposition at nucleation places on the immersed surfaces of Ni mesh. The growth mechanism of NiCo_2_S_4_ thin films by SILAR method is depicted as follows. When Ni(NO_3_)_2_.6H_2_O, Co(NO_3_)_2_.6H_2_O, and Na_2_S.9H_2_O independently dissolved in DDW water, following three reactions occur, respectively.

1 $${\rm{Ni}}{({{\rm{NO}}}_{3})}_{2}.6{{\rm{H}}}_{2}{\rm{O}}\to {\rm{NiO}}+2{{\rm{HNO}}}_{3}+5{{\rm{H}}}_{2}{\rm{O}}$$

2 $${\rm{C}}{\rm{o}}({{\rm{N}}{\rm{O}}}_{3}{)}_{2}.6{{\rm{H}}}_{2}{\rm{O}}\to {\rm{C}}{\rm{o}}{\rm{O}}+2{{\rm{H}}{\rm{N}}{\rm{O}}}_{3}+5{{\rm{H}}}_{2}{\rm{O}}$$

3 $${{\rm{Na}}}_{2}{\rm{S}}+2{{\rm{H}}}_{2}{\rm{O}}\to {{\rm{S}}}^{2-}+2{\rm{NaOH}}$$

When Ni mesh is immersed in the above solution 1, Ni^2+^ ions start adsorbing on the Ni mesh due to attraction between Ni^2+^ ions and the surface of Ni mesh. These forces may be cohesive or van der Waals forces or chemical attractive forces. Similarly, Ni^2+^ adsorbed Ni mesh is immersed in the above solution 2, Co^2+^ ions are adsorbed on Ni mesh. Final step of reaction process was followed by the immersion of Ni^2+^/Co^2+^ coated Ni mesh into Na_2_S anionic solution. During this process, Ni^2+^ and Co^2+^ ions reacts with S^2−^ ions from the Na_2_S anionic solution. Possible reactions are shown below,

4 $${{\rm{H}}}_{2}{\rm{S}}\to {{\rm{HS}}}^{-}+{{\rm{H}}}^{+}\to {{\rm{S}}}^{2-}+{{\rm{H}}}^{+}$$

5 $${{\rm{Ni}}}^{2+}+{{\rm{S}}}^{2-}\to {\rm{NiS}}$$

6 $${{\rm{Co}}}^{2+}+{{\rm{S}}}^{2-}\to \,\mathrm{CoS}$$

In the aqueous solution, Na_2_S dissolves to form S^2−^ ions that are simultaneously hydrolyzed to generate HS^−^ and H_2_S species. These species serve as the sulfur sources for the ion-exchange reaction that converts Ni and Co precursors to form NiCo_2_S_4_. Previously, Dubal *et al*.^25^ have reported similar reaction mechanism for the deposition of Co–Ni mixed hydroxide thin films.

### X-ray diffraction (XRD) analysis

**Figure 1:**
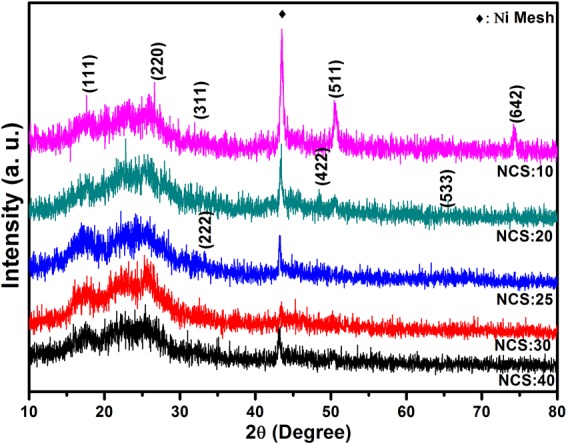
XRD patterns for the NiCo_2_S_4_ thin films prepared with various molar ratios of Ni to Co precursor using SILAR method.

The XRD patterns for the NiCo_2_S_4_ thin films prepared with various molar ratios of Ni to Co source, such as 40:10, 30:20, 25:25, 20:30, 10:40 are shown in Fig. 1. The peaks at 17.54°, 26.60°, 31.85°, 33.06°, 48.05°, 50.53°, 65.42°, and 74.19° are attributed to the (111), (220), (311), (222), (422), (511), (533), and (642) planes of the metallic nickel cobalt sulfide, respectively^27^. For sample NCS:20, two main peaks are observed at 48.05° and 65.42°, which are ascribed to the (422) and (533) planes, corresponding to the ternary phase of the nickel cobalt sulfide. In sample NCS:25 (shown in Fig. 1), one peak was observed at 33.06°, which is in good agreement with the Ni-Co-S^28^. Furthermore, one strong peak at 44.4°, which is originating from the Ni foam^29^. All peaks and peak positions closely match JCPDS card 020-0782, with the synthesized composites exhibiting a cubic crystal structure with lattice parameters a = b = c = 9.30, which closely follows standard results^30,31^. Therefore, it can be concluded that the NiCo_2_S_4_ was successfully deposited on the flexible Ni mesh^31^.

### X-ray photoelectron spectroscopy (XPS) analysis

**Figure 2:**
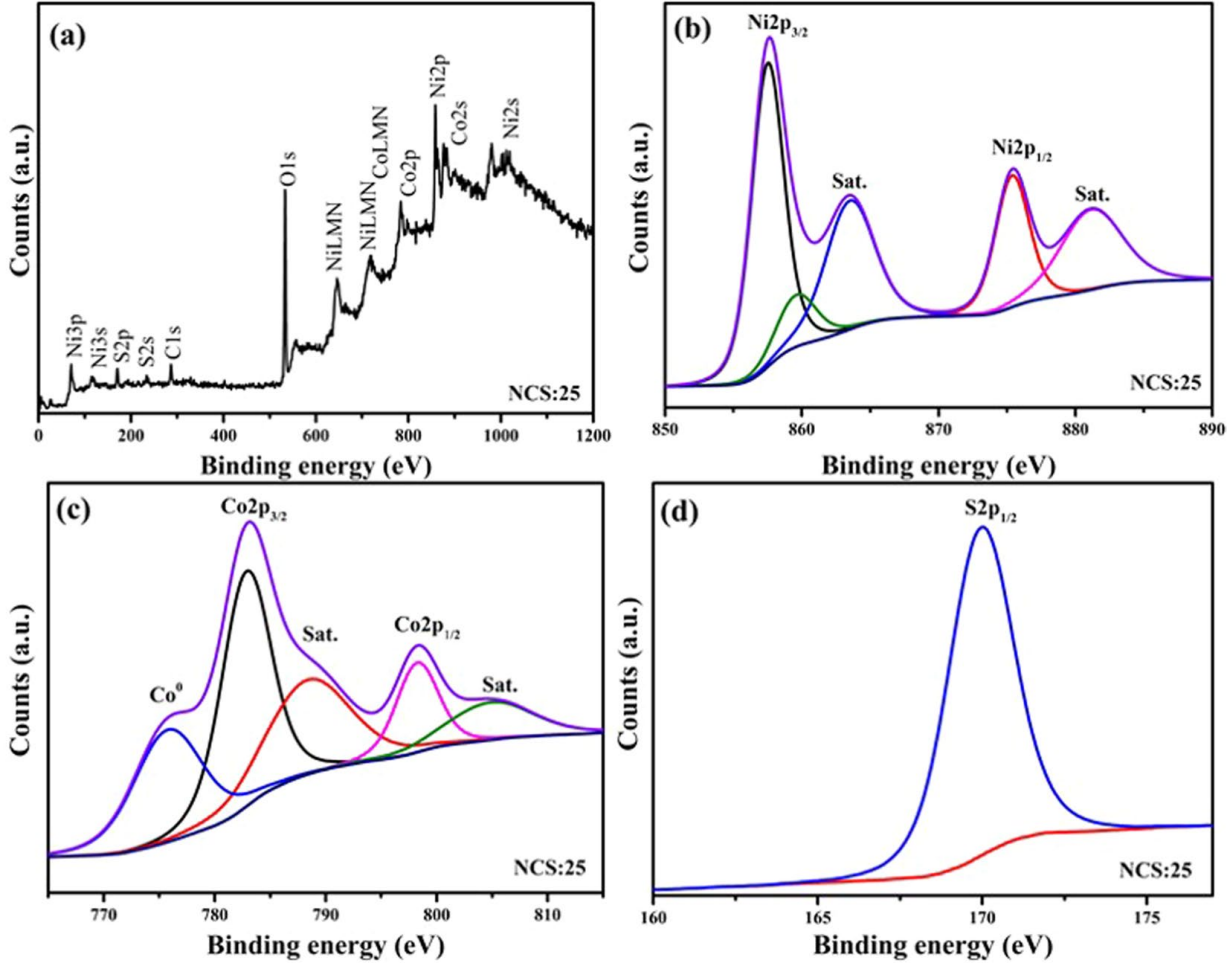
(**a**) Survey spectrum of the optimized NiCo_2_S_4_ sample, (**b**,**c**) Core level spectra for Ni 2p, Co 2p, and S 2p, respectively, at optimized sample NCS:25 thin film on Ni mesh.

XPS was used to further examine the chemical state, elemental valency, and chemical composition of the NiCo_2_S_4_ thin films prepared at optimized molar ratio of Ni and Co. Figure 2a displays the survey spectrum of the NCS:25 sample, which show the presence of Ni, Co, and S elements. Figure 2(a–d) presents the core level spectra for Ni 2p, Co 2p, and S 2p, respectively. The peaks at 168.65 eV correspond to S 2p_1/2_, while the peaks at 857.50 eV and 875.19 eV are related to Ni 2p_3/2_ and Ni 2p_1/2_, respectively^31^. The peak at 533.41 eV is related to oxygen due to exposure to the air^30^. The Co2p spectra exhibited peaks at 783.19 and 798.37 eV, which are attributed to Co 2p_3/2_ and Co 2p_1/2_, respectively. The energy difference between Ni 2p and Co 2p was 17.69 and 15.19 eV, which reflects differences in Ni and Co valence (e.g., Ni^2+^, Ni^3+^, Co^2+^, and Co^3+^)^9,32,33^. For the core-level spectra of S 2p, the peaks at 168.65 eV is related to S 2p_1/2_ (Fig. 2d). From Fig. 2, it can be concluded that Ni^2+^, Co^2+^, Ni^3+^, Co^3+^ and S^2−^ are present in the NiCo_2_S_4_ thin films. The XPS results closely agree with previously reported data for NiCo_2_S_4_ thin films^30,34^.

### Morphological (FE-SEM) analysis

**Figure 3:**
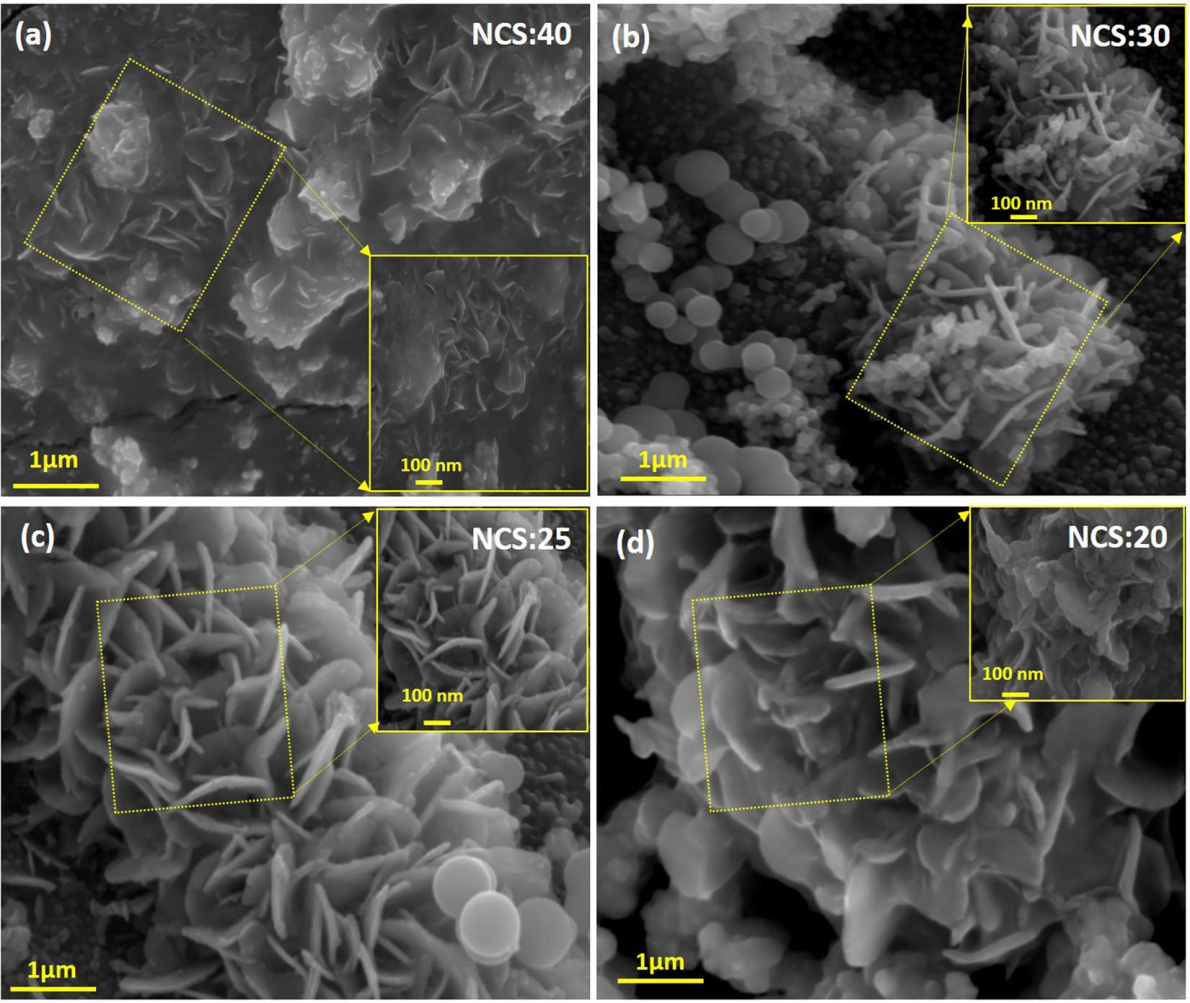
(**a**–**d**) FE-SEM images of NiCo_2_S_4_ thin films prepared for different volume ratios of Ni and Co, like-40:10, 30:20, 25:25, and 20:30, on Ni mesh, respectively and inset show the high magnification of all samples.

**Figure 4:**
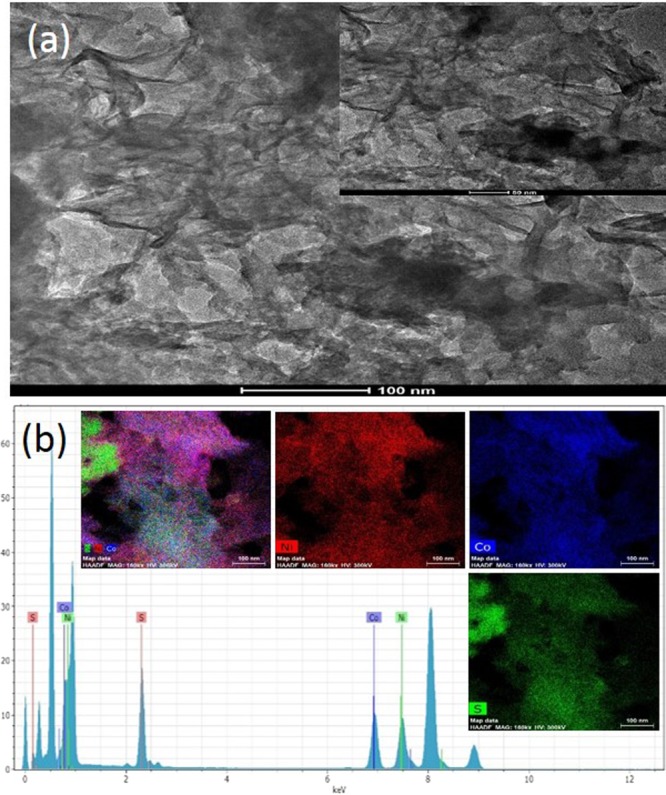
(**a**) TEM, and (**b**) EDS of NiCo_2_S_4_ composites at NCS:25 sample, and inset shows the elemental mapping.

Figures 3(a–d) and S1b present FE-SEM images of NiCo_2_S_4_ thin films prepared for different molar ratios of Ni and Co on Ni mesh, and corresponding high magnification images are shown in the inset. The high magnified image of NCS:25 sample is shown in the Fig. S1b. It can be seen that all of the NiCo_2_S_4_-coated Ni mesh samples were uniformly covered with different types of nanostructure, including petal-shaped structures similar to the top view of a rose, spherical nanoparticles, and interconnected nanoflakes^35–37^. We clearly observed that the nanostructure, depth, thickness, and length of the vertical interconnected nanoflakes were affected by the ratio of Ni to Co. The films fabricated with lower levels of both Ni and Co (i.e., Ni:Co ratios of 40:10 and 10:40) are shown in Figs 3a and S1b. Both NiCo_2_S_4_ thin film samples (Fig. 3(a–d)) exhibited an equal covering of larger-sized nanoplates-like structures and comparatively fewer porous nanostructures^9,38^. In contrast, for a Ni/Co ratio of 25:25, the Ni mesh was completely covered with vertically interconnected nanoflakes in a barrier-wall-like structure (as shown in Fig. 3c). This type of nanostructure provides a large active surface area and faster ion transfer during the electrochemical reaction between the NiCo_2_S_4_ electrode and KOH electrolyte. High scale FE-SEM images of the NiCo_2_S_4_ thin film are also presented in Fig. S1b^31,38^. Compared with the other composites, sample NCS:25 exhibited more hierarchical flake-like inter-network structures, indicating that this sample provides a higher porous surface area for the NiCo_2_S_4_ thin film and higher electrical conductivity^31,39^. The preparation and schematics growth formation of hierarchical NiCo_2_S_4_ thin films on Ni mesh is schematically presented in Fig. S1c^40–42^. In the formation thin films is related to the main four steps, such as nucleation, aggregation, coalescence and growth of nanostructure. Figure 4a shows the transmission electron microscopy (TEM) images of optimized NCS:25 sample. This structure provides a higher surface area because the vertically interconnected nanoflakes were more porous with a thickness and length of 15–20 nm and 80–120 nm, respectively. Vertically interconnected plates are greatly beneficial because they supply both sides of a nanoplates and the ion exchange process is prominently facilitated during cell testing^41,42^. The composition of the nanocomposites was determined using the EDS analysis. The representative EDS spectra of NCS:25 composite is shown in Fig. 4b. The sample shows the presence of Ni, Co and S elements. Figure 4b shows EDS spectrum and inset shows the elemental mapping of NCS:25 sample which confirms formation of a porous nanostructure, respectively^40^. Elemental mapping shows that all the elements are present and homogenously distributed over the film surface for the optimized NCS:25 sample, which was prepared with an equal molar ratios of Ni and Co. Quantitative elemental analysis of nanocomposite revealed that the composition ratio of Ni:Co was in good agreement with stoichiometric ratio. The EDS spectrum indicates that samples are consistent with their elemental signals and stoichiometry is as expected. These results are in good agreement with the XPS results.

### Electrochemical studies

**Figure 5:**
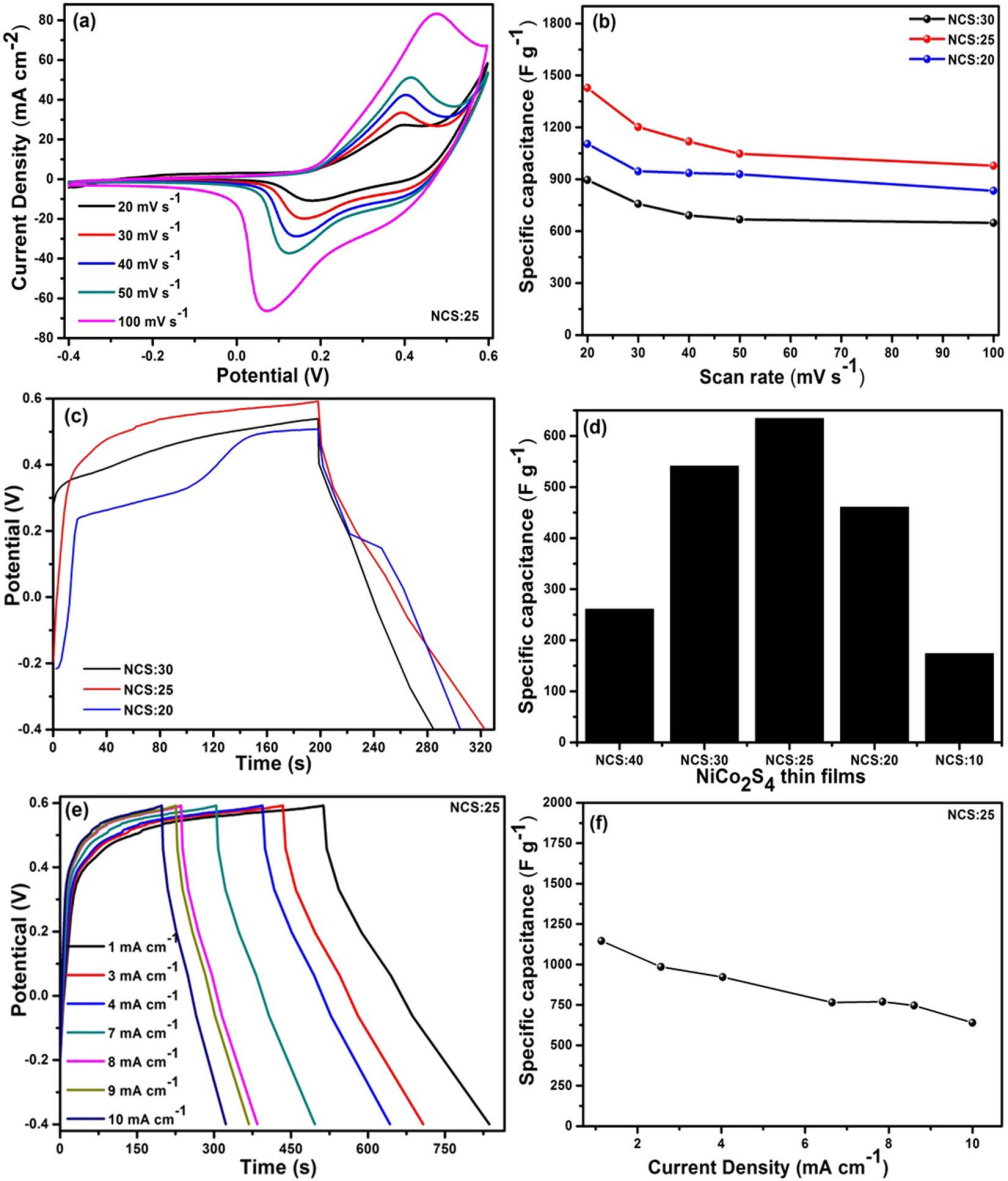
(**a**) CV curves for the optimized NiCo_2_S_4_ composite thin films for various Ni and Co ratios (NCS:25) at scan rate from 20 to 100 mV s^−1^ with a 5 M KOH electrolyte, (**b**) Specific capacitance of NCS:30, NCS:25, and NCS:20 composite thin films, (**c**) Charge-discharge curves for NCS:30, NCS:25, and NCS:20 at 10 mA cm^−2^ current density in 5 M KOH with −0.4 to 0.6 V potential window, (**d**) Specific capacitance for NCS:40, NCS:30, NCS:25, NCS:20 and NCS:10 electrodes, (**e**) Charge-discharge curves for NCS:25 at 1–10 mA cm^−2^ current densities in a 5 M KOH electrolyte, (**f**) specific capacitance of NCS:25 electrode at various current densities from 1–10 mA cm^−2^.

Figures 5a and S2 (supporting information) present the CV curves for the NiCo_2_S_4_ composite thin films for various Ni and Co ratios (NCS:40, NCS:30, NCS:25, NCS:20, and NCS:10) at scan rates from 20 to 100 mV s^−1^ with a 5 M KOH electrolyte. Two redox peaks can be observed in all composite NiCo_2_S_4_ thin films due to their pseudocapacitor behavior and the faradaic reaction^32,43^. The NCS:25 composite (Fig. 5a) demonstrated a higher current density and areal capacitance compared with the other composites due to the vertically interconnected nanoflakes providing a higher surface area and faster ion exchange and faradaic reactions^31,32,39,44^. The higher current density indicates that the NiCo_2_S_4_ thin films shows a higher specific capacitance compared with other composites. The NCS:25 electrodes exhibited the best electrical properties compared with the other four samples because it promoted higher electrical conductivity and faster electron transport and was more porous^30,31,45^. The redox reaction showed the most intense peaks of the NiCo_2_S_4_ thin films due to the presence of different Ni^2+^ and Co^2+^ valencies in the KOH electrolyte^7^.

7 $$\mathrm{CoS}\,+{{\rm{OH}}}^{-}\to {\rm{CoSOH}}+{{\rm{e}}}^{-}$$

8 $${\rm{CoSOH}}+{{\rm{OH}}}^{-}\to \,\mathrm{CoS}\,+{{\rm{H}}}_{2}{\rm{O}}+2{{\rm{e}}}^{-}$$

9 $${\rm{NiS}}+{{\rm{OH}}}^{-}\to {\rm{NiS}}({\rm{OH}})+{{\rm{e}}}^{-}$$

Figures 5b and S3 shows the specific capacitance of NCS:40, NCS:30, NCS:25, NCS:20, and NCS:10 for various scan rates from 20–100 mV s^−1^. The specific capacitance was calculated using the following Eq. 10^46,47^:

10 $${C}_{s}=\frac{1}{mv({V}_{c}-{V}_{a})}{\int }_{{V}_{a}}^{{V}_{c}}I(V)dV$$

where, C_S_ is the specific capacitance (F g^−1^), (V_c_-V_a_) is the potential window, m is the mass of the electrode, and v is the scan rate. The specific capacitance were found 570, 896, 1427, 1106, and 790 F g^−1^ for NCS:40, NCS:30, NCS:25, NCS:20, and NCS:10, respectively, indicating that the NCS:25 electrode was most suitable for supercapacitor applications due to its superior electrochemical performance. Figures 5a and S2(b,c) shows CV curves of NCS:30, NCS:25 and NCS:20 electrodes with different scan rates from 20–100 mV s^−1^. The sample NCS:25 shows the highest specific capacitance values with 20 mV s^−1^ scan rate. The values of specific capacitance decreased with an increase in the scan rate: 1427, 1202, 1118, 1046, and 976 F g^−1^ for a scan rate of 20, 30, 40, 50 and 100 mV s^−1^, respectively. Several previously reported NiCo_2_S_4_ based electrodes and their supercapacitor performance are tabulated in the Table 1. It indicates that the NiCo_2_S_4_ thin films synthesized by chemical SILAR method and vertical interconnected flakes like nanostructure shows the better performance.

**Table 1:** NiCo_2_S_4_ based electrodes and their electrochemical performance.

Material	Substrate	Synthesis	Sp. Capacitance	Morphology	Ref.
Ni–Co–S	Carbon	Electrochemical	1418 F g^−1^ at 5 A g^−1^	Nanosheet	^2^
Ni–Co–S	Graphene Foam	Electrochemical	2918 F g^−1^ at 1 A g^−1^	Nanosheet	^3^
NiCo_2_S_4_	Ni foam	Hydrothermal	738 F g^−1^ at 4 A g^−1^	Nanotube	^7^
NiCo_2_S_4_	Ni foam	Hydrothermal	800 F g^−1^ at 12 mA cm^−2^	Nanorod	^8^
NiCo_2_S_4_	Ni foam	Micelle confined	1304 F g^−1^ at 2 A g^−1^	Nanosheet	^10^
NiCo_2_S_4_	Ni foam	Solvothermal	1870.2 F g^−1^ at 2 A g^−1^	Core-shell hollow spheres	^11^
NiCo_2_S_4_	Ni foam	Solvothermal	1,036 F g^−1^ at 1.0 A g^−1^	hollow spheres	^12^
CoNi_2_S_4_	Ni foil	Electrodeposition	1999 mF cm^−2^ at 2 mA cm^−2^	Flower	^14^
Co_x_Ni_(3-x_) S_2_/Go	Ni foam	Hydrothermal	15.6 F g^−1^ at 10 mA cm^−2^	Core/Shell	^51^
NiCo_2_S_4_/C	Ni foam	Hydrothermal	1093 F g^−1^ at 0.5 A g^−1^	Nanosheets	^19^
Ni–Co–S	Ni foam	Chemical	1406.9 F g^−1^ at 0.5 A g^−1^	Nanosheet	^20^
Ni-Co-S@Ni-W-O	Ni foam	Hydrothermal	1988 F g^−1^ at 2 A g^−1^	Nanosheets	^21^
Ni_1.5_Co_1.5_S_4_	Ni foam	Solvothermal	1093 F g^−1^ at 1 A g^−1^	Nanoparticles	^22^
Ni_3-x_Co_x_S_4_	Carbon cloth	Hydrothermal	1859 F g^−1^ at 1 A g^−1^	Nanotube	^23^
Ni_0.25_Co_0.75_ S_4_	Ni foam	Anion-exchange reaction	676 C g^−1^ at 1 A g^−1^	Sea Urchin	^24^
CoNi_2_S_4_	Carbon nanofiber	Electrospinning, electrodeposition	181 F g^−1^ at 0.1 A g^−1^	Nanoparticles	^52^
Ni-Co-S	Ni foam	Solvothermal	852 F g^−1^ at 4 A g^−1^	Hollow Nanocolloids	^53^
NCS/rGO/CNT	Ni foam	Hydrothermal	1102 F g^−1^ at 1 A g^−1^	Hierarchical layered	^54^
NiCo_2_S_4_	Ni mesh	SILAR	1427 F g^−1^ at 20 mV S^−1^	Interconnected nanoflakes	In present work

Figures 5c and S4 present the charge-discharge curves for NCS:40, NCS:30, NCS:25, NCS:20, and NCS:10 at current density of 10 mA cm^−2^ in a 5 M KOH electrolyte, respectively. The specific capacitance was calculated using the following Eq. 11^48^:

11 $${C}_{S}=\frac{{I}_{d}\times {T}_{d}}{\Delta V\times m}$$

where, C_S_ is the specific capacitance (F g^−1^), T_d_ is the discharge time (s), m is the active mass of electrode, I is the current (mA), and ΔV is the potential window. From Fig. 5d, we observed that sample NCS:25 shows the higher specific capacitance at 10 mA cm^−2^, due to the interconnected and highly porous nanostructures and fast ions transferred from electrodes and electrolyte. Figure 5e displays the charge-discharge curves of NCS:25 electrode at different current densities vary from 1–10 mA cm^−2^ at 5 M KOH electrolyte. The specific capacitance of NCS:25 were calculated as 1146, 985, 922, 776, 764, 745 and 640 F g^−1^ at current densities of 1–10 mA cm^−2^ (shown in Fig. 5f).

Figure S5 represents the capacitance retentions of the NCS:25 as a function of the number of GCD cycles at 10 mA cm^−2^ in 5 M KOH electrolyte solution. The cycling test shows an increase in specific capacitance after initial 500 cycles which is due to activation followed by a gradual decrement in next 1000 cycles. More than 90% capacity retention after 2000 cycles, demonstrate high stability and high performance of the electrode. In summary, after the CV, charge-discharge analyses, and capacitance retention test, the NCS:25 composite was demonstrated to be the superior electrode material when compared with binary sulfides^26,39^.

### Electrochemical impedance spectroscopy (EIS)

**Figure 6:**
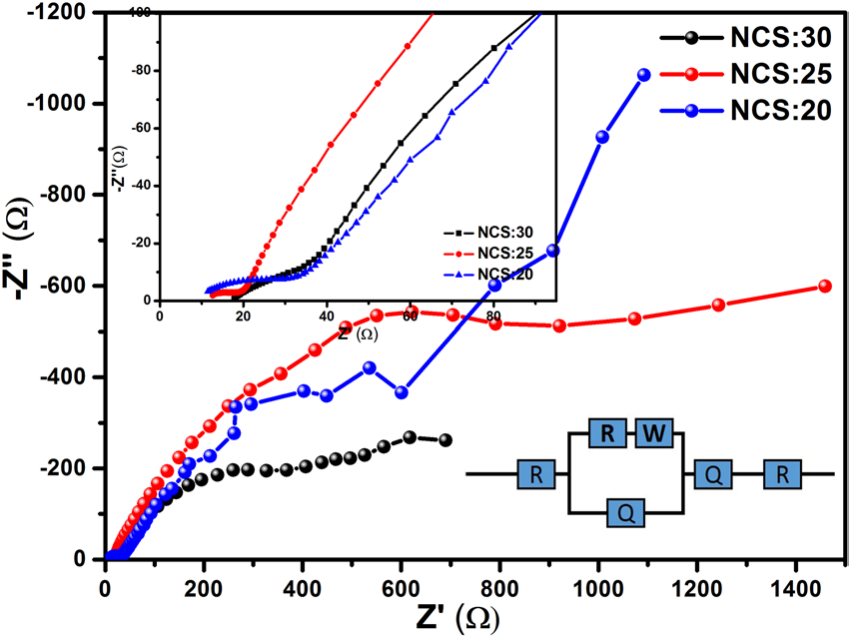
Nyquist plots for the NCS:30, NCS:25, and NCS:20 electrodes at 5 M KOH electrolyte. (Inset: Proposed equivalent circuit for EIS fitting).

EIS was used to determine the electrical properties of the NiCo_2_S_4_ samples prepared at different Ni/Co ratios. Figures 6 and S6 show the Nyquist plots for the NCS:40, NCS:30, NCS:25, NCS:20, and NCS:10 electrodes. All of the samples were considered to determine the solution resistance (R_S_), charge transfer resistance (R_ct_), Warburg impedance (W), and double-layer capacitance (C). The R_S_ are 15.4, 14.65, 10.54, 11.78, and 24.59 Ω and R_ct_ are 83.2, 38.02, 20.00, 31.82, and 322.26 Ω for NCS:40, NCS:30, NCS:25, NCS:20, and NCS:10, respectively. The proposed equivalent circuit is shown in inset of Fig. 6. The measured values of solution resistance indicate that the NCS:25 shows the lower value as compared to the other electrodes, it means NCS:25 electrode shows high electrical conductivity as compared to the other electrodes. The NCS:25 composite thin film also depicted a lower charge transfer resistance than the other four electrodes. These results indicate that the NiCo_2_S_4_ thin films had a lower solution and charge transfer resistance, which may be due to their high electrical conductivity and highly porous nanoflakes-like nanostructure^46–50^.

## Conclusion

In summary, 3D nanoflakes-like NiCo_2_S_4_ thin films were successfully synthesized using the SILAR method on Ni mesh with different molar ratios of Ni and Co. The specific capacitance results demonstrated lower performance by NCS:40 and NCS:10 due to their compact morphology, while NCS:25, with its vertically interconnected nanoflakes morphology, produced the highest specific capacitance (1427 F g^−1^ at 20 mV s^−1^). We thus successfully developed a new approach for the fabrication of Ni thin-film architecture and demonstrated that NiCo_2_S_4_ electrodes are promising materials for supercapacitor applications.

## Supplementary information

Supporting information
