# Supplementary material for: Novel approach to synthesize NiCo2S4 composite for high-performance supercapacitor application with different molar ratio of Ni and Co
Source: Sci Rep. 2019 Sep 23;9:13717. doi: 10.1038/s41598-019-50165-5 (PMC6757066; doi:10.1038/s41598-019-50165-5)
Supplement: Supplementary file 1 — Supporting information [file 41598_2019_50165_MOESM1_ESM.docx]

**Supporting information**

**Novel approach to synthesize NiCo_2_S_4_ composite for high-performance supercapacitor application with different molar ratio of Ni and Co.**

S. K. Shinde^1^, Sivalingam Ramesh^2^, C. Bathula^3^, G. S. Ghodake^1^, D. -Y. Kim^1^, A. D. Jagadale^4^, A. A. Kadam^5^, D. P. Waghmode^6^, T. V. M. Sreekanth^7^, Heung Soo Kim^2^, P. C. Nagajyothi^7*^,

H. M. Yadav^8*^

*^1^Department of Biological and Environmental Science, Dongguk University-Ilsan, Biomedical Campus, Goyang-si, Gyeonggi-do, 10326, South Korea*

*^2^Department of Mechanical, Robotics and Energy Engineering, Dongguk University Seoul, 04620, South Korea.*

*^3^Division of Electronics and Electrical Engineering, Dongguk University Seoul, 04620, South Korea.*

*^4^Department of Electrical and Electronics Engineering, SASTRA Deemed University, Thanjavur 613401, Tamilnadu, India*

*^5^Research Institute of Biotechnology and Medical Converged Science, Dongguk University, Biomedi Campus, Ilsandong-gu, Goyang-si, Gyeonggi-do 10326, South Korea.*

*^6^Department of Chemistry, Sadguru Gadage Maharaj College, Karad, 415124.*

*^7^College of Mechanical Engineering, Yeungnam University, Gyeongsan 48135, South Korea.*

*^8^Department of Energy and Materials, Engineering Dongguk University Seoul, 04620, South Korea.*

***Corresponding author:**

**1. Prof. H. M. Yadav**

Department of Energy and Materials Engineering, Dongguk University Seoul, 04620, South Korea

E-mail: hemrajy@gmail.com

***Co-Corresponding author:**

2. **Professor P.C. Nagajyothi**

College of Mechanical Engineering, Yeungnam University, Gyeongsan 48135, South Korea.

E-mail: pcnnagajyothi@gmail.com

**Figure S1** (a) Schematic experimental setup of SILAR method, (b) FE-SEM images of NiCo_2_S_4_ thin films prepared for molar ratios of Ni and Co (10:40) on Ni mesh, and (c) Schematics growth formation of NiCo_2_S_4_ thin films on Ni mesh using SILAR method.

**Figures S2 (a-d)** CV curves for the NiCo_2_S_4_ composite thin films for various Ni and Co ratios (NCS:40, NCS:30, NCS:20, and NCS:10) at scan rates from 20 to 100 mV s^-1^ with a 5M KOH electrolyte.

**Figure S3** Specific capacitance of NCS:40 and NCS:10 composite thin films with various scan rates from 20-100 mV s^-1^.

**Figure S4** Charge-discharge curves of NCS:40, and NCS:10 at 10 mA cm^-2^ current density in a 5M KOH electrolyte.

**Figure S5** Cycling test of NCS:25 at 10 mA cm^-2^ current density in a 5M KOH electrolyte.

**Figure S6** Nyquist plots of the NCS:40, and NCS:10 electrodes at 5 M KOH electrolyte.

**
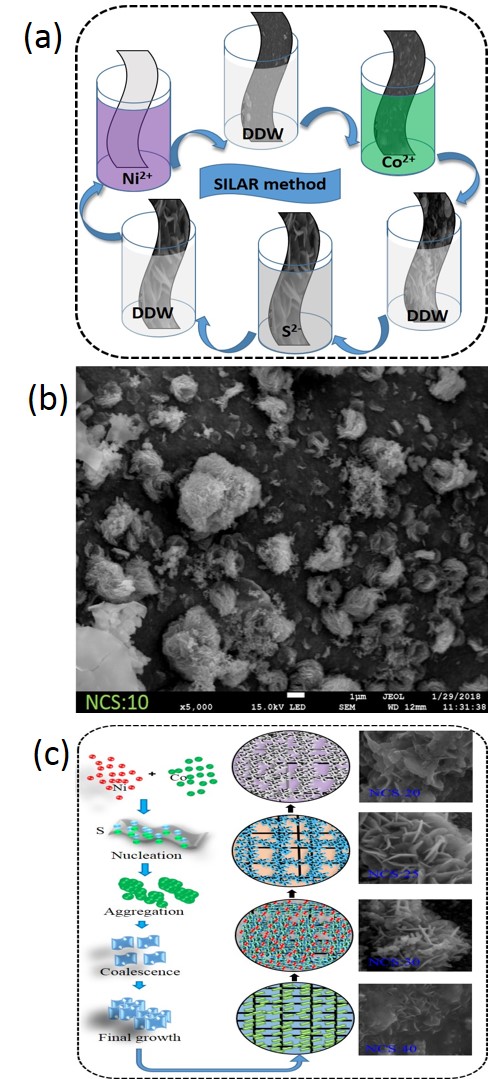
**

**Figure S1**

**
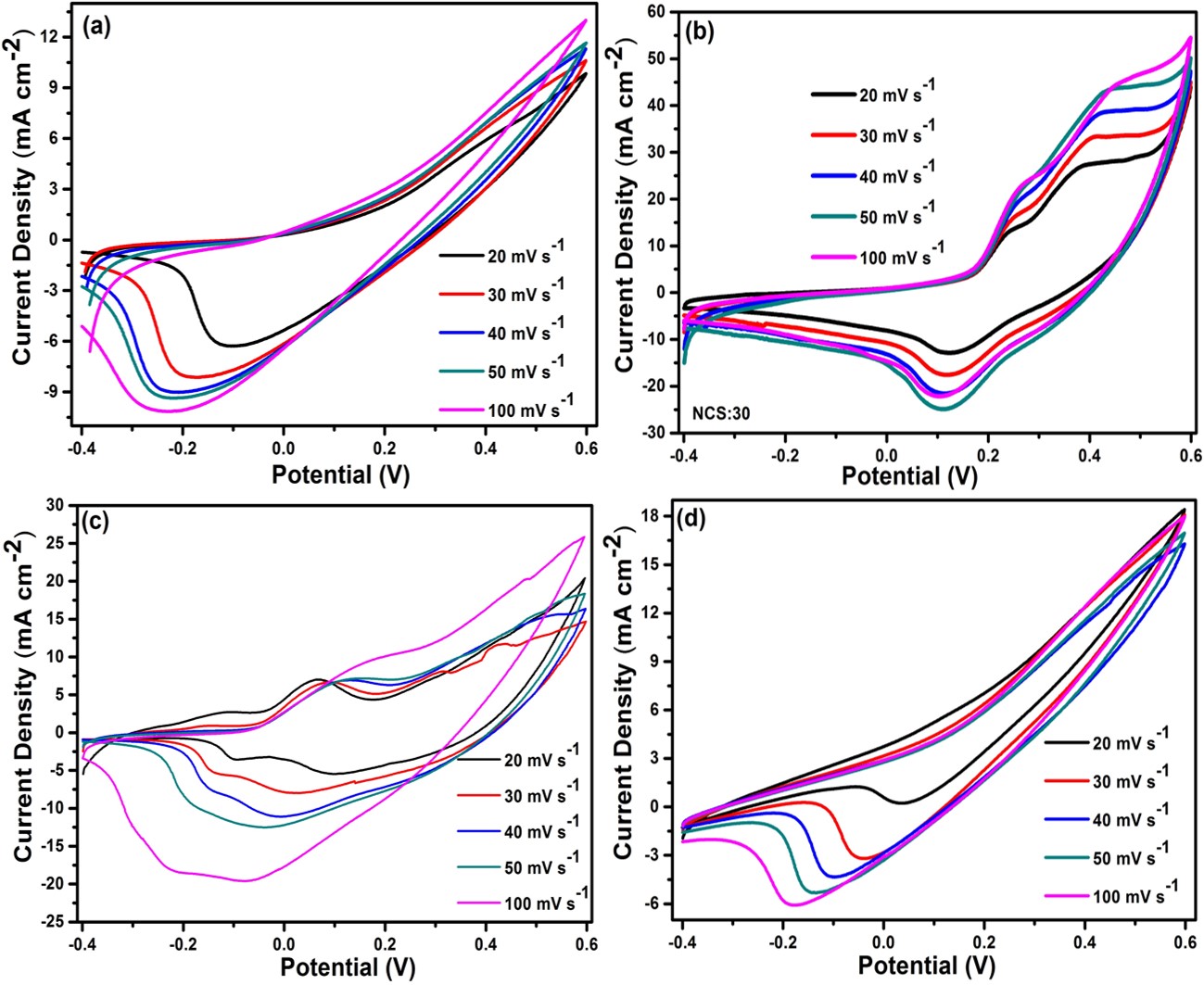
**

**Figure S2**

**
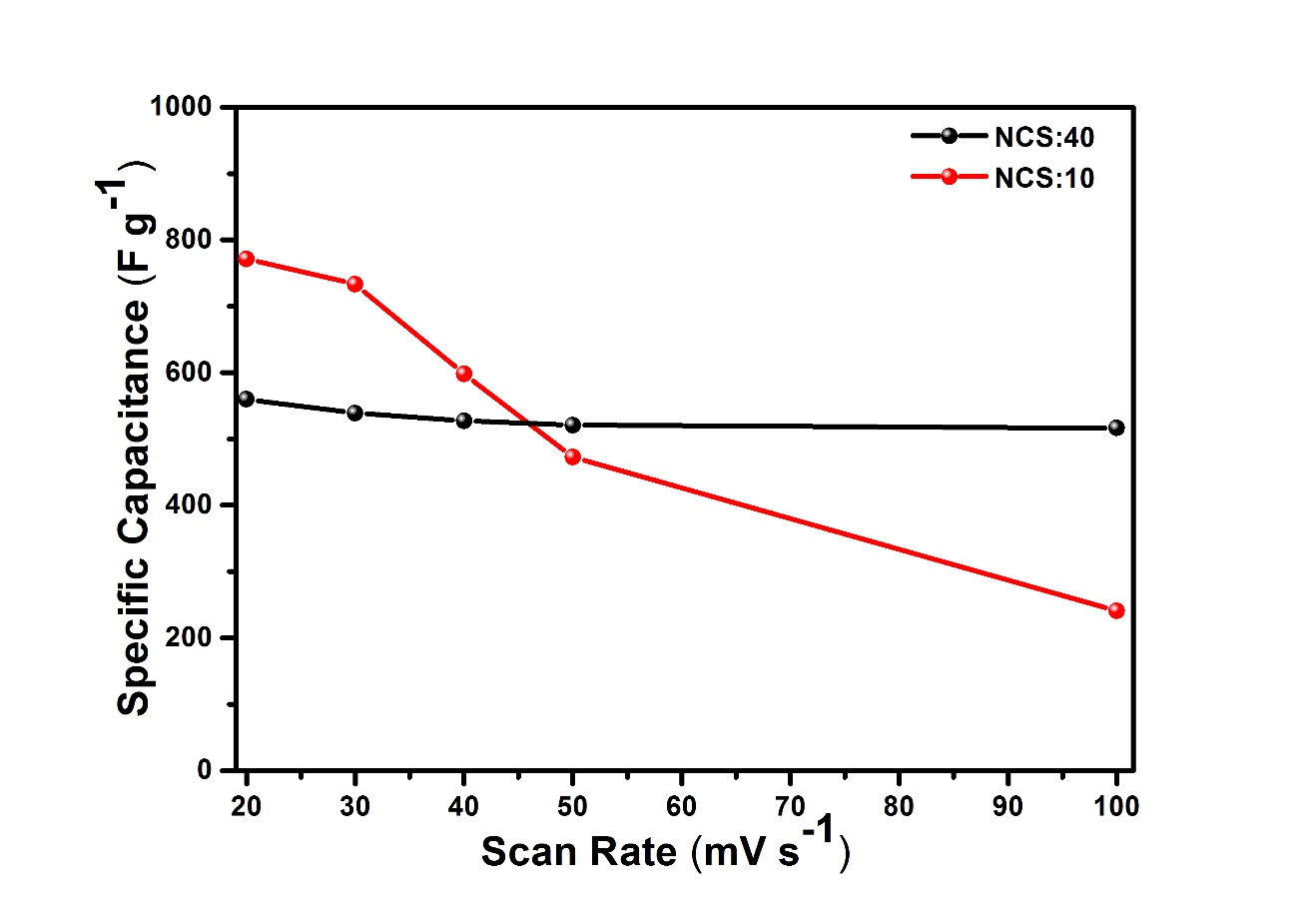
**

**Figure S3**

**
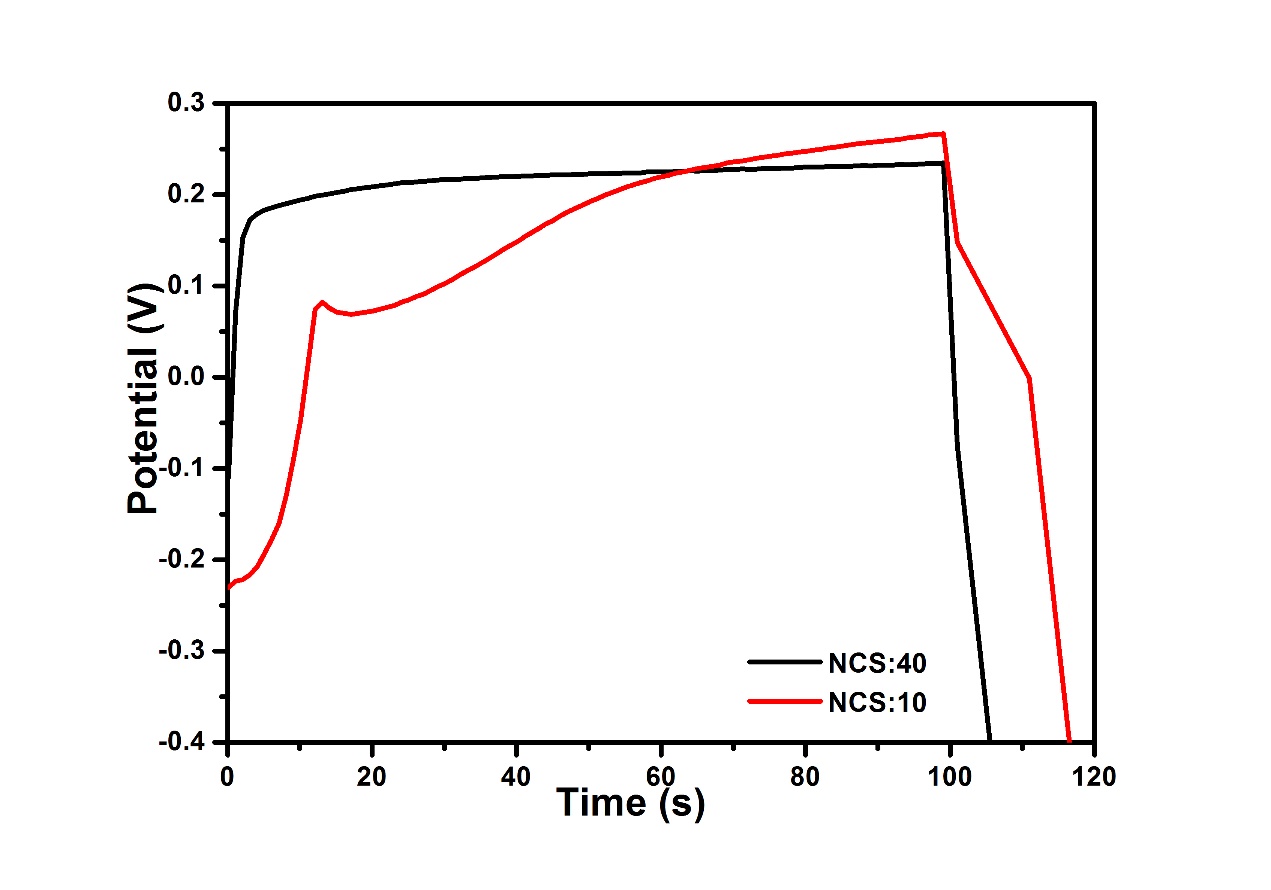
**

**Figure S4**

**
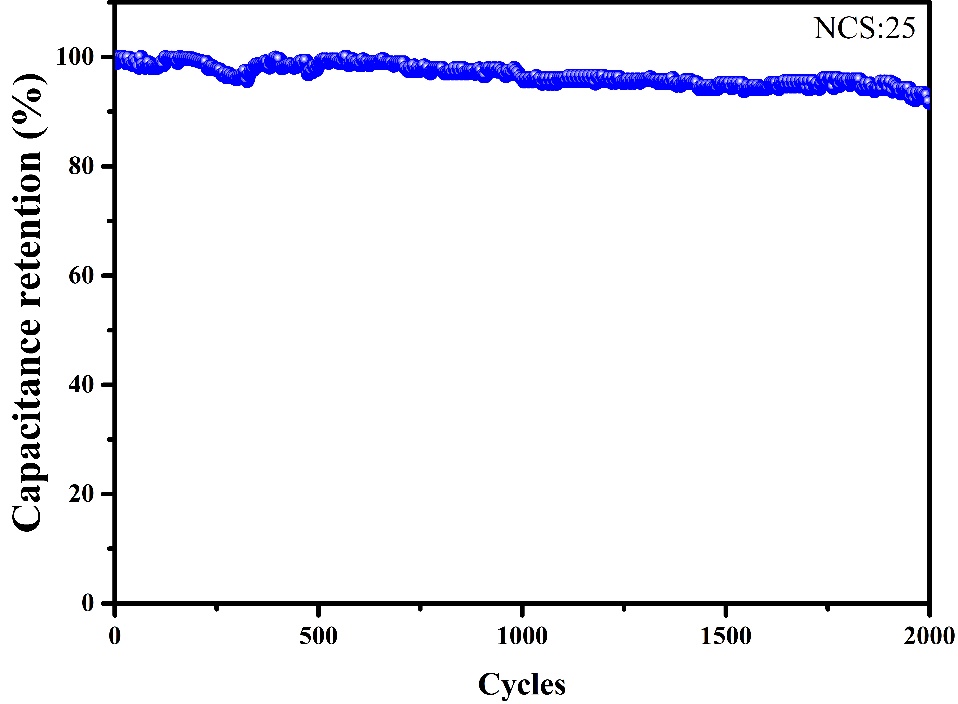
**

**Figure S5**

**
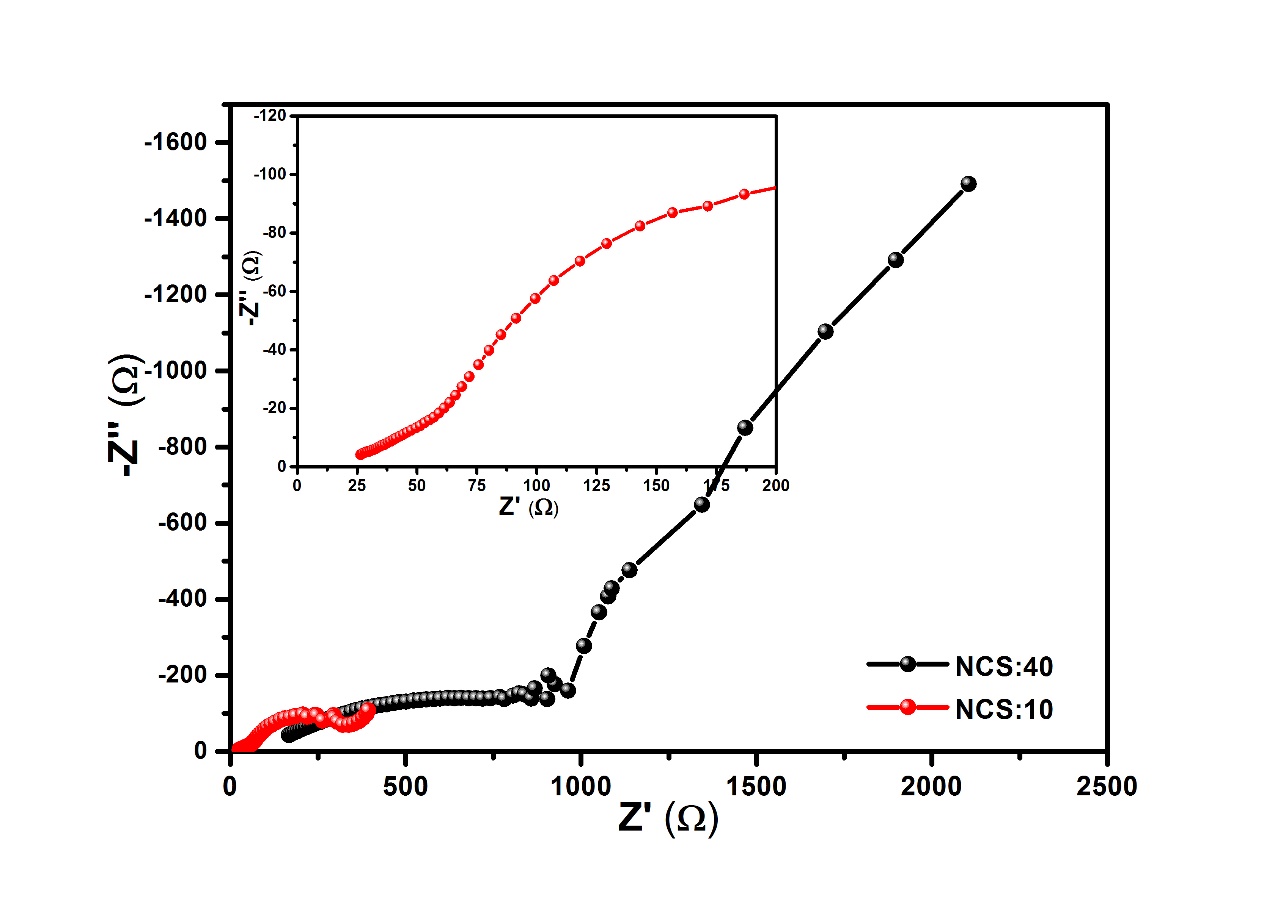
**

**Figure S6**
